# The Impact of Exercise Play on the Biomechanical Characteristics of Single-Leg Jumping in 5- to 6-Year-Old Preschool Children

**DOI:** 10.3390/s25020422

**Published:** 2025-01-13

**Authors:** Zhanbing Song, Bojie Hou, Zhongqiu Ji, Guiping Jiang

**Affiliations:** College of P.E. and Sports, Beijing Normal University, Beijing 100875, China; 201931070018@mail.bnu.edu.cn (Z.S.); hbjrlrw@163.com (B.H.); jizhongqiu61@bnu.edu.cn (Z.J.)

**Keywords:** exercise play, motor development, human simulation, preschool children, single-leg jump

## Abstract

Objective: This study aimed to investigate the effects of a 12-week self-designed exercise game intervention on the kinematic and kinetic data of the supporting leg in preschool children during the single-leg jump. Methods: Thirty 5- to 6-year-old preschool children were randomly divided into an experimental group (EG) and a control group (CG). The BTS SMART DX motion capture analysis system was used to collect single-leg jump data before the intervention. The experimental group underwent a 12-week intervention, with self-designed exercise games conducted three times a week for 30 min each session, while the control group only participated in regular kindergarten recess activities and physical education classes. After the intervention, the same equipment was used to collect single-leg jump data again, and the kinematic and kinetic data were analyzed using Anybody 7.4 simulation software. Results: After the intervention, the experimental group showed significant changes in joint angles and joint torques, with a notable increase in the force exerted by dominant muscles such as the vastus medialis, vastus lateralis, and gastrocnemius and a significant increase in the ground reaction force. Although the control group also showed some changes in the dominant muscles, the changes were not as significant as those in the experimental group. Conclusions: A 12-week exercise game intervention significantly improved the technique and force characteristics of 5- to 6-year-old preschool children during the single-leg jump, making muscle exertion more focused and efficient and effectively enhancing explosive power and performance during the single-leg jump.

## 1. Introduction

Early childhood (ages 3–6) is a critical period for motor development, significantly influencing future physical competence, activity levels, and overall health. Mastering fundamental motor skills during this stage facilitates the acquisition of complex movements later in life and promotes lifelong physical well-being [1,2,3]. Research shows that preschool children with well-developed motor skills experience fewer difficulties when learning advanced movements in adulthood and are more likely to maintain a healthy weight and lifestyle [2,4]. However, many children in early childhood education settings do not engage in sufficient physical activity to support optimal development [5].

Existing studies often focus on general motor activities or isolated exercises [6,7,8,9], relying primarily on subjective assessment tools such as developmental scales and observational checklists to evaluate motor skills [10,11,12]. Although these approaches offer valuable insights, they may lack the precision required to detect subtle biomechanical changes occurring during motor development. To address this gap, the present study utilized motion biomechanics analysis to quantify the kinematic and kinetic changes in preschool children following a 12-week game-based intervention. By employing the BTS SMART DX motion capture system and Kistler force platforms, this study provides objective, high-resolution data on joint angles, muscle forces, and ground reaction forces, offering a comprehensive understanding of motor development. 

Game-based interventions specifically targeting challenging motor tasks, such as single-leg jumps, remain underexplored. Single-leg hopping, a continuous movement performed on one foot, is considered a fundamental yet demanding skill for preschool children. It serves as a moderate-to-high-intensity activity that promotes motor development and fulfills daily physical activity needs [13]. The integration of quantitative biomechanical analysis in this research not only enhances the reliability of the findings but also enables precise tracking of intervention outcomes, distinguishing it from traditional observational methods.

Single-leg hopping is an important action for assessing children’s motor development. Among all basic motor skills, the single-leg hop is relatively difficult for preschool children to perform. This action requires the coordination of multiple muscle groups, and through single-leg hopping, the development levels of lower limb muscle strength, coordination ability [2], and balance ability [14] in young children can be reflected. Professor Li Jing’s research indicates that the development of single-leg hopping ability in children aged 3–10 is relatively slow [15]. The current research on single-leg hopping mainly focuses on the characteristics of the jump, stiffness, and sex differences. Natsuki Sado and colleagues, through the analysis of differences between single-leg and double-leg jumps in 10 male subjects, found that leg extension was more complete during single-leg jumps and that the height of the swinging leg had a significant impact on the jump height [16]. Previous studies have shown that single-leg hops are often used to test joint stiffness because they are not disturbed by vertical rebound. Control of joint stiffness has been proven to be closely related to running, jumping performance, and sports injuries [17]. Beerse et al.’s study found that during single-leg hopping, children had weaker control over horizontal movements compared with adults [18]. Research also indicates that single-leg hopping in children aged 5–11 is still in the development stage, and lower limb stiffness changes with frequency [19]. Some researchers have focused on sex differences in vertical jumping during early childhood and concluded that there are no sex differences in vertical jumping at this age [20]. Based on the above research, we find that studies on single-leg jumps often focus on the analysis of the movement itself, while the issues of improving athletic performance in single-leg jumps and enabling students to quickly master the basic technique of single-leg jumping remain urgent problems that need to be addressed.

In summary, this study conducted a 12-week intervention with self-designed games for 5- to 6-year-old children to improve their physical activity abilities and promote their motor development. The kinematic and kinetic differences before and after the intervention were analyzed to explore the impact of the game scheme on the children’s single-leg hopping performance. The research hypotheses were as follows: (1) The 12-week self-designed game intervention will result in superior performance in single-leg hopping. (2) The 12-week game intervention will improve the efficiency of children’s movements during single-leg hopping.

## 2. Participants and Methods

### 2.1. Participants

Children aged 5–6 from a private kindergarten in Renqiu City who voluntarily participated in the experiment and met the test conditions were selected. With the help of teachers, the participants were informed about the experimental conditions. Children with limited activity ability, limb or organ diseases, or unstable health conditions were excluded. A total of 30 children were included in the study. Before the game intervention, the participants were randomly divided into a control group (CG) and an experimental group (EG), with 15 children in each group. After the 12-week exercise game intervention, the experimental group consisted of 15 children. During the data analysis process, 5 children in the control group showed severe anomalies, resulting in only 10 preschool children having valid data. This study was approved by the Ethics Committee of the Faculty of Psychology at Beijing Normal University, review number: 201910210061. The specific details of the test were communicated to the parents and teachers of the participating children.

### 2.2. Methods

#### 2.2.1. Exercise Game Intervention and Control

Before the intervention, students were randomly divided into an experimental group and a control group. During the 12-week intervention, without interfering with the normal teaching activities of the experimental group, the experimental group received an exercise game intervention three times a week for 30 min each session during recess. The control group only participated in the regular kindergarten recess exercises and a weekly physical activity class. All children in the experimental group had to participate in the game intervention three times a week for 30 min each session. Children who did not meet this condition were not included in the analysis. During the intervention, the head teachers and assistant teachers of each experimental class were selected as intervention implementers. Before the intervention, researchers and intervention teachers practiced each game to ensure that the main movements of the exercise games were fully taught to the students. The self-designed exercise game content is shown in Table 1.

#### 2.2.2. Data Collection

Before the intervention, key morphological indicators were measured for 30 children, including height, weight, head and torso height, head width, distance between the ankle and hip, knee width, leg length, hip depth, and pelvis width. The BTS SMART DX infrared optical motion capture analysis system, 8 high-speed cameras, and 22 reflective markers were used to accurately collect the participants’ three-dimensional data. Kinematic data were captured using the BTS SMART DX infrared optical motion capture system with a sampling frequency of 100 Hz. The marker point information is shown in Table 2.

The mechanical data analysis was conducted through synchronized data collection from the Kistler force platform and BTS SMART DX motion capture system, followed by inverse dynamics analysis using Anybody 7.4 software. The Kistler platform recorded ground reaction forces (GRFs) at 1000 Hz, capturing vertical, horizontal, and lateral forces during jumps and landings. Simultaneously, the BTS SMART DX system, operating at 100 Hz with eight infrared cameras and 22 markers, tracked three-dimensional motion and joint angles. The systems were synchronized via an external trigger, ensuring temporal alignment with an error margin of less than 1 ms. The collected data were imported into Anybody 7.4, where participant-specific lower limb models were created. Inverse dynamics calculations applied Newton–Euler equations to derive joint torques at the hip, knee, and ankle by integrating GRF and motion data. Muscle forces were estimated using a musculoskeletal model that distributed loads across key muscles, such as the quadriceps and gastrocnemius, through optimization algorithms. To ensure accuracy, pre-experiments identified and reduced errors caused by movement instability, and outliers exceeding two standard deviations were excluded during the final data analysis. The results provided joint torque profiles, muscle force outputs, and energy transfer metrics.

The BTS SMART DX system provided angular resolution up to 0.01°, ensuring high precision in tracking joint movements. However, occasional marker occlusion may introduce minor discrepancies. These discrepancies were minimized through a rigorous calibration and verification process, which involved repeated measurements and manual corrections using the Motion Capture System SMART DX 700 (Milano, Italy). After completing these calibrations, the optimized Anybody 7.4 software was used for parameter optimization before data analysis, to minimize marker errors. The optimization process was considered successful when the difference between the marker points and the system model’s marker points was less than 0.01°, further enhancing system precision. In the experiment, we also used a high-precision force platform with a sampling frequency of 1000 Hz, capable of recording 1000 force values per second, ensuring high-precision capture of force signals during rapid movements. To ensure the accuracy of the force platform, we conducted static zero calibration, static load calibration, and dynamic load calibration before each experiment, along with zero and span calibration. To improve the reliability of the force data, we performed repeated measurements of the force platform and applied data smoothing algorithms to process instantaneous data, reducing noise interference.

After the intervention, the participation of children in the experimental group was recorded, and the results showed that all children met the intervention conditions. The infrared data of all children in the experimental and control groups were collected during the single-leg jump, following the pre-intervention measurement steps.

Single-leg jump: Start from a natural standing position with feet together and core tightened. Choose one foot as the support foot (e.g., right foot), while the other foot (left foot) is lightly lifted off the ground. Keep the body upright with shoulders and hips aligned. Then, push off the ground forcefully with the supporting leg, using the right foot to push down and propel the body upward quickly. In the air, keep the body vertical, legs together and as straight as possible and arms swinging naturally to help maintain balance.

**Figure 1:**
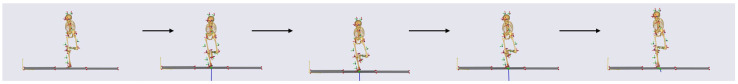
Figure of children’s lateral sliding step motion.

In this study’s analysis, the moment the supporting leg bent downward was defined as the start of the action, and the moment the toes of the supporting leg left the ground was defined as the end of the action (Figure 1).

#### 2.2.3. Data Analysis

By importing the data collected by the BTS CAPTURE infrared capture software into the Anybody 7.4 software, we selected the frame numbers for one cycle of the single-leg jump performed by the children and adjusted the lower limb gait model based on their morphological data. Through system parameter optimization, kinematic, and inverse dynamic analysis, we obtained the joint angles, joint torques, muscle forces, and ground reaction force data for the supporting-side lower limb during one movement cycle.

This study primarily analyzed the kinematic and dynamic characteristics of the supporting-side lower limb. Therefore, the collected data included the joint angles and joint torques of hip flexion, hip abduction, hip external rotation, knee flexion, ankle plantar flexion, and subtalar joint eversion on the supporting side. The muscles collected included the rectus femoris (RF), vastus medialis (VM), vastus lateralis (VL), biceps femoris (BF), semitendinosus (SED), semimembranosus (SEB), gastrocnemius (GAT), soleus (SL), tibialis anterior (TA), gluteus maximus (GMa), gluteus medius (GMe), and gluteus minimus (GMi) on the supporting side. Additionally, the ground reaction force during the single-leg jump process for the supporting leg was included.

#### 2.2.4. Statistical Analysis

In this study, data processing was performed using Python 3. To eliminate the influence of weight differences among different students on the results, muscle force, joint torque, and ground reaction force for all participants were normalized by body weight. In the analysis of significant differences between the experimental and control groups before and after the intervention, normally distributed data were analyzed using repeated measures ANOVA, while non-normally distributed data were analyzed using the Wilcoxon signed-rank test.

Due to the non-normal distribution of some data, the Wilcoxon signed-rank test was used to analyze differences in non-normally distributed data in this study. Additionally, to ensure data quality, the interquartile range (IQR)-based method was employed to filter outliers, excluding data points that exceeded two standard deviations. This approach ensured the robustness and reliability of the analysis results.

## 3. Results

### 3.1. Joint Angles

**Figure 2:**
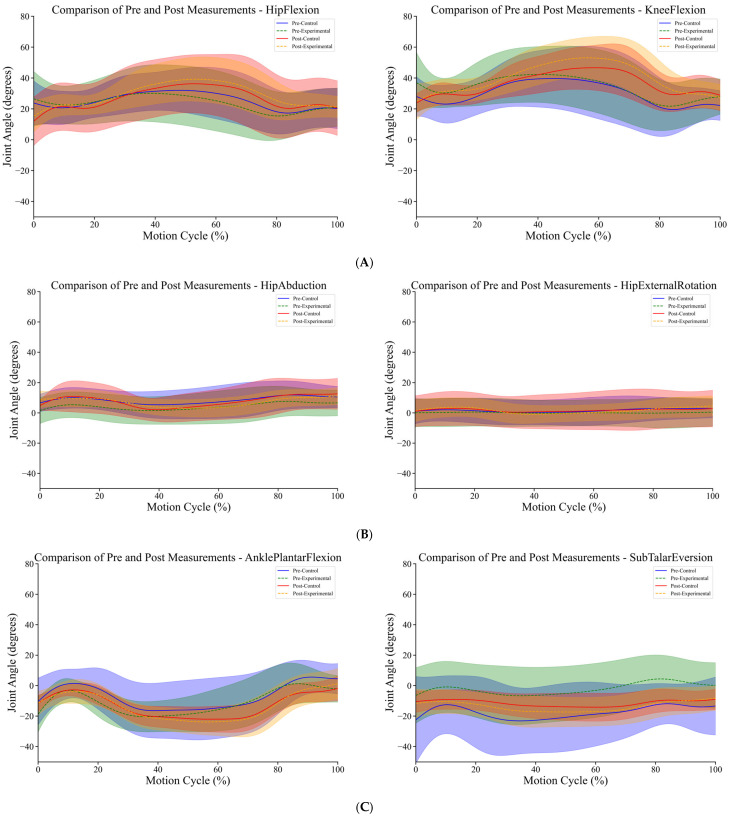
(**A**) Diagram of joint angle changes in hip flexion and knee flexion during single-leg jumps before and after intervention. (**B**) Diagram of joint angle changes in hip abduction and hip external rotation during single-leg jumps before and after intervention. (**C**) Diagram of joint angle changes in ankle plantar flexion and subtalar eversion during single-leg jumps before and after intervention. The shaded area represents the range of standard deviation.

As shown in Figure 2, the trend of ankle plantar flexion angle during the single-leg jump process was similar between the control and experimental groups for 5-year-old children. However, the experimental group exhibited an earlier change point in the plantar flexion angle before the intervention. Before the intervention, the experimental group reached the maximum ankle plantar flexion angle at approximately 30% of the movement, after which the angle began to decrease. After a period of game intervention, there was a noticeable delay in the plantar flexion angle change point for the experimental group, which may be related to more effective push-off during the jump.

According to Table 3, after the intervention, the increase in the ankle plantar flexion angle in the control group was greater compared with the experimental group. As shown in Figure 2, after a period of teaching intervention, the experimental group exhibited an increase in hip angle during the 0–50% and 80–100% phases of the movement. In contrast, the hip abduction angle in the control group showed a similar trend before and after the intervention. Table 3 shows that after the intervention, there was a significant increase in the hip abduction angle in the experimental group, while there was no significant difference in the hip angle for the control group before and after the intervention. As shown in Figure 2B, after a period of teaching intervention, the hip external rotation angle in the experimental group significantly increased during the single-leg jump, particularly during the 0–30% and 70–100% phases of the movement. In contrast, there was no significant change in the hip external rotation angle in the control group, and the trend of hip external rotation during the entire jump process remained consistent before and after the intervention. According to Table 3, after the teaching intervention, the hip flexion angle during the single-leg jump significantly increased in the experimental group, with a greater increase compared with the control group. During the complete movement, the experimental group reached the maximum hip flexion angle at 60% of the action, which then began to decrease. Additionally, the rate of decrease in hip flexion angle in the experimental group after the intervention was higher than in other groups. According to Table 3, the increase in knee flexion angle after the intervention was similar between the experimental and control groups. However, Figure 2A reveals that students who underwent teaching intervention reached the peak knee flexion angle earlier during the single-leg jump, at approximately 60% of the movement. In contrast, the peak knee flexion angle for the control group occurred later, around 70% of the movement, and was lower than that of the experimental group. Based on the charts, we found that although there were some differences in knee flexion angles between the experimental and control groups before the intervention, the overall knee flexion movement patterns were similar (Figure 2B and Table 3). According to the charts, both the control and experimental groups showed significant changes in the subtalar joint eversion angle after the intervention. However, the subtalar joint eversion angle significantly decreased in the control group, while it significantly increased in the experimental group. Additionally, before the teaching intervention, the experimental group exhibited some inversion of the subtalar joint at approximately 80% of the complete movement (Figure 2C and Table 3).

### 3.2. Muscle Force

**Figure 3:**
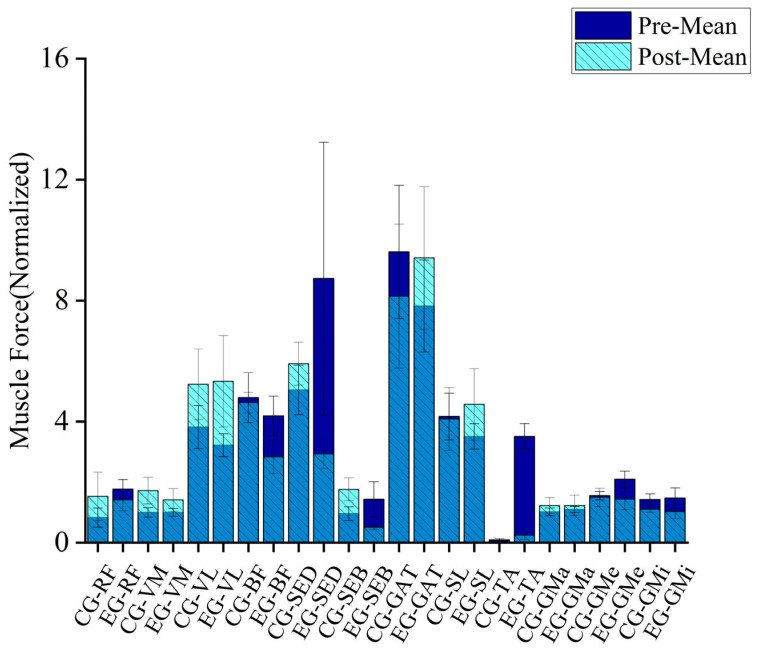
Diagram of muscle changes in the supporting-side lower limb during single-leg jumps before and after intervention.

As shown in Table 4 and Figure 3, after 13 weeks of game-based intervention, there were significant changes in muscle force during the single-leg jump task among the students. In the experimental group, the activation of the rectus femoris significantly decreased, while in the control group, it significantly increased. Both groups showed a significant increase in the activation of the vastus medialis after the intervention, with the control group exhibiting a larger increase. The activation of the vastus lateralis significantly increased in both groups after the intervention, with the increase being more pronounced in the experimental group compared with the control group. The activation of the semitendinosus significantly decreased in the experimental group after a certain period of intervention, while it significantly increased in the control group. A similar pattern was observed for the semimembranosus. The activation of the gastrocnemius and soleus significantly increased in the experimental group after the intervention during the jump, whereas in the control group, the activation of these muscles decreased. The activation of the tibialis anterior significantly decreased in the experimental group after the intervention, while no significant change was observed in the control group. Before and after the intervention, the activation of the gluteus maximus increased in both the experimental and control groups, although not significantly. Regarding the gluteus medius and gluteus minimus, the experimental group exhibited reduced muscle force during the single-leg jump after the intervention, while the control group also showed significant changes, although to a lesser extent.

### 3.3. Joint Torque

**Figure 4:**
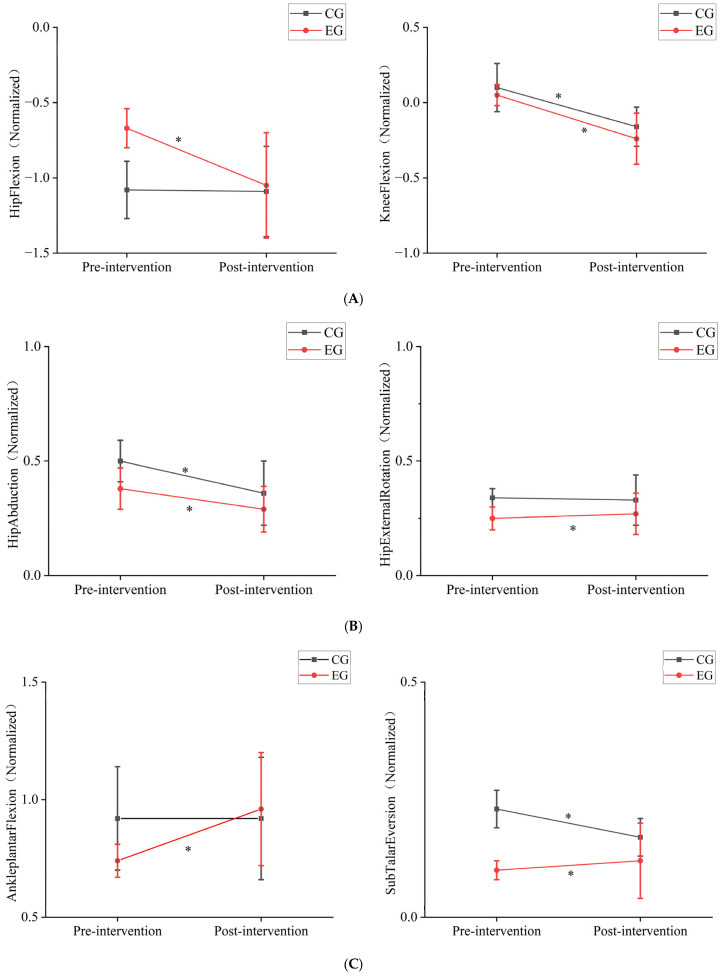
(**A**) Diagram of joint torque changes in hip flexion and knee flexion during single-leg jumps before and after intervention. (**B**) Diagram of joint torque changes in hip abduction and hip external rotation during single-leg jumps before and after intervention. (**C**) Diagram of joint torque changes in ankle plantar flexion and subtalar eversion during single-leg jumps before and after intervention. * indicates a *p*-value < 0.01 for post-intervention vs. pre-intervention data.

As shown in Table 5 and Figure 4, the 12-week game intervention significantly affected the joint torques in the experimental group. After the intervention, the joint torque for hip abduction in the experimental group significantly decreased, while the joint torques for hip flexion, hip external rotation, knee flexion, ankle plantar flexion, and subtalar joint eversion showed varying degrees of increase, with the increases being quite significant (*p* < 0.05). In contrast, the control group showed a significant decrease in joint torques for hip flexion and subtalar joint eversion, while the joint torques for hip flexion, hip external rotation, and ankle plantar flexion did not show significant changes. In both the control and experimental groups, the knee joint torque values shifted from positive to negative before and after the intervention, indicating that the movement strategies during the single-leg jump changed for both groups.

### 3.4. Ground Reaction Force (GRF)

**Figure 5:**
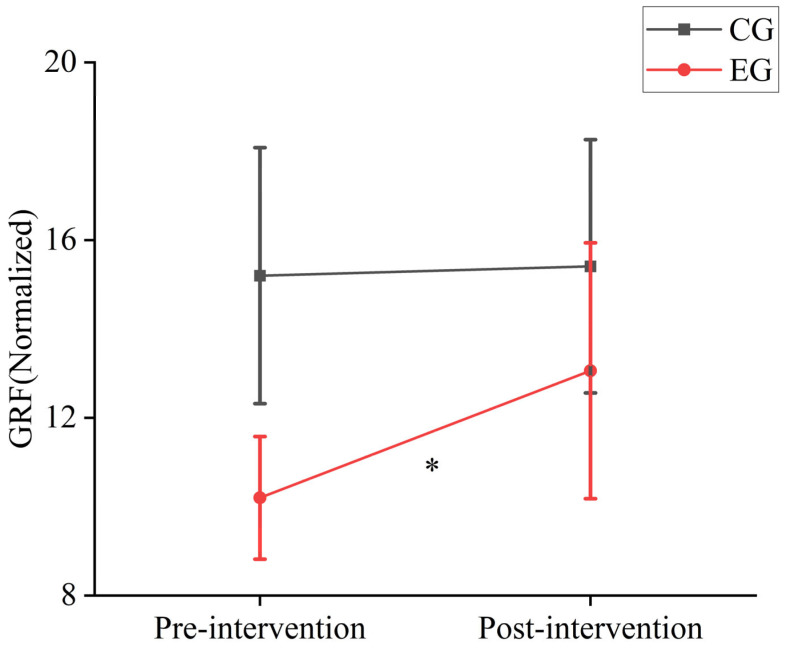
Diagram of ground reaction force changes during single-leg jumps before and after intervention. * indicates a *p*-value < 0.01 for post-intervention vs. pre-intervention data.

As shown in Figure 5, after a period of game-based intervention, the experimental group exhibited a significant increase in ground reaction forces, while the control group did not show significant changes in ground reaction forces during the single-leg jump before and after testing.

Following the 12-week intervention, the experimental group exhibited significant improvements in key kinematic and kinetic parameters during single-leg jumps (Table 3 and Table 4). Specifically, joint mobility and coordination were enhanced, with noticeable increases in hip flexion, knee flexion, and ankle plantar flexion. Muscle force data, normalized by body weight, showed increased activation in key muscle groups such as the vastus lateralis and gastrocnemius, contributing to greater ground reaction forces during take-off.

Comparative analysis revealed that the experimental group, which engaged in 12 distinct exercise-based games, achieved greater improvements in joint stability and muscle activation compared with the control group, which continued with regular physical activities without targeted interventions (Figure 2 and Figure 3).

These findings suggest that multi-movement, game-based interventions are more effective in enhancing lower limb strength and coordination than general physical activity alone.

## 4. Discussion

### 4.1. The Impact of Exercise Game Intervention on Lower Limb Joint Mechanical Characteristics in Children

This study investigated the effects of a 12-week custom game-based intervention on the development of single-leg jump performance in 5- to 6-year-old children. Peter et al. discovered that during the jumping process, both the hip and knee joints play equally important roles in the jump, but children utilize the hip joint less effectively compared with adults [21]. Panchao Zhao et al. emphasized in their study on the impact of aging on preschool children’s vertical jump performance that hip joint mobility is crucial for effective jumping, and they recommended incorporating hip joint exercises into game activities [22]. In this study, a 12-week game-based intervention led to significant improvements in hip abduction and external rotation abilities during single-leg jumps in the experimental group, while no significant changes were observed in the control group’s hip abduction and external rotation angles (Table 3). Muscle force analysis (Table 4) revealed that in the experimental group, the activation of the gluteus minimus and gluteus medius, which primarily control hip abduction, significantly decreased after the intervention. Conversely, the gluteus maximus, which controls hip external rotation, showed a slight increase in activation, although not significantly different before and after the intervention. Additionally, the experimental group showed a significant decrease in hip abduction moment and a significant increase in hip external rotation moment after the intervention. This suggests that the 12-week game-based intervention allowed the children in the experimental group to achieve greater hip abduction and external rotation angles with reduced muscle effort during the jump. In contrast, the control group experienced a decrease in hip abduction angle during the jump, which led to reduced muscle strength in the primary muscles (gluteus medius and gluteus minimus), resulting in lower joint moments for these muscles. Figure 2B illustrates that in the experimental group, the increase in hip abduction angle after intervention was most pronounced during the 0–50% and 70–100% phases of the jump. This indicates that the intervention enhanced the stability of the supporting-side hip joint and improved the efficiency of the gluteus medius and gluteus minimus during the eccentric phase of the jump, thereby reducing motion deformation caused by instability of the center of mass. Furthermore, the increase in hip abduction and external rotation angles provided the participants with a greater range of motion during the jump, thereby enhancing the flexibility of the movement. During the latter half of the jump, the increase in the hip abduction angle helped maintain hip stability during the push-off phase, improving jump efficiency and force transfer. The decrease in hip abduction moment may indicate reduced unnecessary force output and decreased load on the abductor muscles, thus reducing fatigue in the hip abductor muscles (gluteus medius and gluteus minimus) and enhancing movement efficiency (Table 5). The increase in hip external rotation moment also suggested that the game-based intervention altered the control strategy for single-leg jumps. This enhancement in hip external rotation moment further stabilized the hip joint and pelvis, reduced unnecessary internal rotation, and minimized body tilt and rotation, thus improving the overall movement effectiveness.

During a single-leg jump, hip flexion helps students lower their center of gravity during the preparation phase, providing adequate conditions for the subsequent push-off phase. In this study, after a 12-week game-based intervention, the hip flexion angle in the experimental group significantly increased. Although the control group also experienced significant changes in hip flexion angle, the changes were relatively smaller compared with the experimental group. Additionally, based on changes in joint moments (Table 5), we found that after 12 weeks of intervention, the hip extension moment in the experimental group significantly increased, while there were no significant changes in the joint moment for the control group. Combining muscle force data before and after the intervention (Table 4), we observed a significant decrease in the strength of the primary hip flexor muscle (rectus femoris) in the experimental group, while the strength of the adductor and vastus lateralis muscles significantly increased. This suggests that the intervention may have optimized the movement pattern in the experimental group, enhancing muscle coordination and resulting in an increased hip flexion angle. The strength of the hip extensor muscles (gluteus maximus and hamstrings) in the experimental group showed a decreasing trend, yet the extension moment increased during the movement. This may be due to improved neuromuscular control, which optimized movement coordination. In the control group, the increase in hip flexor muscle force after the intervention was greater compared with the experimental group; however, the increase in hip flexion angle was not as significant as in the experimental group. This suggests that the control group did not achieve optimized muscle work efficiency for hip flexion despite the increased muscle force. In the control group, the increase in hip extensor muscle force was inconsistent after the intervention, and there was no significant change in the hip extension moment. This indicates that without intervention, the movement strategy for hip extension during single-leg jumps did not undergo significant changes. Soest et al. found that during single-leg jumps, the hip joint moment was higher compared with double-leg jumps in professional volleyball players [23]. This finding is consistent with the results of our study.

By analyzing the changes in hip joint moments, joint angles, and primary muscle force related to hip flexion, abduction, and external rotation, we found that after 12 weeks of game-based intervention, the experimental group exhibited a significant increase in hip joint angles during single-leg jumps, and muscle strength efficiency also significantly improved. These changes indicate that following the game-based intervention, children in the experimental group showed significant improvements in both the stability of their movements and the efficiency of the jumping process during single-leg jumps.

Previous studies have shown that knee flexion and extension during single-leg jumps are crucial phases of the take-off process [22,23]. Additionally, more efficient eccentric movements at the start of the jump can generate higher concentric output during the jump phase, thereby enhancing performance [24,25,26]. This study found that after the intervention, both the control and experimental groups of children showed a significant increase in knee flexion angle during single-leg jumps. Based on joint moment data, we found that after the intervention, both the control and experimental groups exhibited an “increasing” trend in knee flexion moments during single-leg jumps. Specifically, the average knee flexion moments of both groups shifted from positive to negative values. According to Table 5, the knee flexion moments of both groups were predominantly positive before the intervention but turned negative afterward, with the experimental group showing a particularly significant change. This indicates that after 12 weeks of intervention, both groups shifted from producing knee flexion moments to producing knee extension moments during single-leg jumps. According to muscle force data (Table 4), after the intervention, the experimental group showed a significant increase in the activation of the vastus medialis and vastus lateralis muscles, with the increase in the vastus lateralis being particularly notable, while the strength of the rectus femoris decreased. This suggested that the intervention significantly improved the stability of the medial and lateral sides of the knee during the jump in the experimental group. Previous research also indicates that the vastus lateralis is a key muscle for vertical jumps in children [22], and the effective use of knee extensor muscles is considered crucial for achieving mature vertical jumping [27]. After the intervention, the experimental group experienced a significant decrease in the strength of the biceps femoris, semitendinosus, and semimembranosus muscles during the jump. These muscles are primarily responsible for knee flexion. The decrease in muscle strength led to a reduction in knee flexion moments, while the significant increase in strength of the vastus lateralis and vastus medialis may be the main reason for the shift from knee flexion to knee extension moments. Compared with the experimental group, the control group showed different changes after 12 weeks. The control group, which did not undergo intervention, exhibited significant increases in the strength of the knee extensor muscles (rectus femoris, vastus lateralis, and vastus medialis) and also showed significant increases in the strength of the semitendinosus and semimembranosus muscles. However, there was no significant change in the strength of the biceps femoris. These changes are related to the physical activities the children engaged in at school. The muscle changes in the experimental group were similar to those in the control group, with a relatively larger knee extension moment in the experimental group. This suggests that the 12-week intervention may not have directly affected the knee extensor muscles or the posterior muscles like the semitendinosus and semimembranosus, but it did enable students to produce greater force output during the jump, improve muscle work efficiency, and enhance the upward driving force.

Although the knee joint is the primary energy-generating joint during jumping, studies have also shown significant differences in ankle joint dynamics among athletes of different levels during vertical jumps [23,28]. According to Table 5, after 12 weeks of intervention, the average values of ankle plantar flexion angles in both the control and experimental groups were negative, indicating a higher proportion of ankle dorsiflexion during the movement. Both groups showed a significant increase in the average values of ankle dorsiflexion angles, indicating a greater range of motion in the ankle joint during single-leg jumps. Based on joint moment data, after the intervention, the experimental group showed a significant increase in ankle plantar flexion moments, which were higher than those in the control group. Additionally, the strength of the gastrocnemius and soleus muscles in the experimental group also significantly increased, with the gastrocnemius showing the most notable change (Table 4). Research by Soest et al. has shown that during single-leg jumps, the gastrocnemius muscle is often at a higher activation level [23]. The lack of significant changes in muscle force for these muscles in the control group is a key reason for the increased joint moments in the experimental group. This feature also suggests that the 12-week game-based intervention had a significant positive impact on ankle joint force output during single-leg jumps. Based on subtalar joint eversion data (Table 5), after 12 weeks of intervention, the experimental group showed a negative value for subtalar joint eversion angles, indicating that both groups exhibited higher and significantly increased subtalar joint inversion angles during take-off. Before the intervention, the experimental group had smaller subtalar joint inversion angles compared with the control group, indicating a smaller range of ankle motion in the experimental group. After 12 weeks of game-based intervention, the experimental group showed a significant increase in subtalar joint inversion angles, suggesting that the range of motion of the subtalar joint increased, and the ankle joint’s flexibility improved, providing more directional options during the jump. The experimental group exhibited a significant increase in subtalar joint inversion angles, indicating enhanced control of foot inversion during the jump. In contrast, the control group showed decreased inversion angles, suggesting reduced flexibility in the subtalar joint during the jump. Additionally, the decrease in joint moments in the control group implied that the experimental group experienced reduced rotational moments at the foot during the jump, leading to a decreased burden on the foot and ankle and improved energy concentration and work efficiency. Although the experimental group showed an increase in subtalar joint inversion angles, the subtalar joint eversion moments did not change significantly, indicating that related muscles (e.g., tibialis anterior) may have adapted to the new range of motion through improved strength and control, maintaining stable moments. This suggests an enhancement in muscle coordination and control in the experimental group, allowing them to maintain moment stability at greater inversion angles.

### 4.2. The Impact of Exercise Game Intervention on Ground Reaction Forces in Children

Ground reaction force is an important physical quantity that describes the interaction between the body and the ground, reflecting the mechanical characteristics of contact with the ground during movement. Irineu et al. argued that an increase in ground reaction force directly impacts jump height in vertical jumps [29]. This study found that after 12 weeks of game-based intervention, the experimental group showed significant increases in joint angles during single-leg jumps, with particularly noticeable changes in hip flexion angles. This suggests that the experimental group used sufficient hip flexion to provide a better preparatory position for subsequent push-off, facilitating smoother force transmission. After 12 weeks of intervention, there were significant differences in muscle force between the experimental and control groups during single-leg jumps. The experimental group showed a marked decrease in muscle force data post-intervention, with the main increase in muscle force observed in the gastrocnemius and vastus lateralis muscles. Previous studies have also indicated that these muscles are dominant in single-leg jumps [22,23]. Although the overall muscle force in the experimental group showed a downward trend, the ground reaction force exhibited a significant increase (Figure 5). This indicates that the experimental group’s overall movement strategy changed after 12 weeks of game-based intervention, with more focused muscle force and a notable improvement in muscle work efficiency. In contrast, the control group showed a continuous increase in lower limb muscle force during single-leg jumps, but there was no significant change in ground reaction force. This suggests that the control group’s movement strategy was unstable, with more dispersed muscle force and lower energy efficiency.

## 5. Conclusions

The 12-week custom game-based intervention effectively enhanced the experimental group’s selection and coordination of movement strategies during single-leg jumps. The intervention significantly optimized joint moments and angles at the ankle, knee, and hip during take-off, resulting in clearer muscle force distribution and more focused and coordinated muscle force. Additionally, the experimental group showed a significant increase in ground reaction force during single-leg jumps. These changes in kinematic and dynamic data indicate that the 12-week intervention markedly improved lower limb strength, movement continuity, and stability in 5- to 6-year-old preschool children during single-leg jumps. This study demonstrates that a 12-week game-based intervention can effectively enhance lower limb strength and performance in single-leg jumps and significantly optimize coordination and execution efficiency.

## Figures and Tables

**Table 1:** Self-designed game table.

Game Name	Purpose of the Game	Method of the Game
Dribbling relay	Ball dribbling ability	Groups are divided, standing at both ends with a 10 m gap, performing a face-to-face dribbling relay.
Tug of war	Enhances coordination of large muscle groups in the upper and lower limbs	Teams are randomly divided into two groups for a tug of war competition based on the number of participants.
Jump rope relay	Practice single- or double-leg jumping ability	Groups are divided, standing at both ends with a 15 m gap, performing a running jump rope relay.
Chase and run	Develops lower limb strength and body control ability	In pairs, one person stands on one foot while lifting the other foot, and they chase each other.
Accuracy kicking	Develops kicking ability	Kicking balls of different colors into the goal, with the size of the goal randomly changing during the exercise.
Look at the sky from behind	Develops rolling and crawling ability	Facing the mat, bending the body to look at the sky between the legs, with protective assistance, subtly introducing forward roll practice.
Accuracy throwing	Develops upper limb throwing ability	Throwing sandbags into a basket from different distances.
My paper airplane flies the furthest	Develops coordination between upper limb throwing and lower limb coordination	Throwing paper airplanes made during craft class in a flying competition during break time, seeing whose airplane flies the farthest.
I can hang on	Practice gripping ability	Hanging on the horizontal bar competition, with assistance from the teacher to flip over.
Tail pulling	Practice acceleration and directional running ability	Using tails of different colors for grouping, pulling each other’s tails while protecting one’s own tail.
Accuracy hitting	Practice hitting balls with hands	Self-throwing and self-hitting a volleyball to see who can hit the balloon into the designated goal.
Distance hitting	Practice controlling hitting ability	After forming groups, practice hitting a baseball for distance.

**Table 2:** The placement information of marker points.

Placement of Marker Points	Number
C7 vertebra	1
Left/right acromion	2
Left/right anterior superior iliac spine	2
Midpoint between left and right posterior superior iliac spines	1
Left/right greater trochanter	2
Left/right mid-thigh	2
Left/right lateral tibial condyle	2
Left/right fibular head	2
Left/right mid-calf	2
Left/right lateral malleolus	2
Left/right heel	2
Left/right fifth metatarsal	2

**Table 3:** Average joint angle for the complete cycle during single-leg jumps before and after intervention.

Joint Angle (Degree)	Pre-CG (Mean ± Std)	Post-CG (Mean ± Std)	Pre-EG (Mean ± Std)	Post-EG (Mean ± Std)
Hip flexion	25.14 ± 4.97	26.80 ± 6.56 *	23.66 ± 4.57	28.90 ± 7.11 *
Hip abduction	8.48 ± 2.11	7.81 ± 3.43	4.35 ± 1.99	7.06 ± 2.77 *
Hip external rotation	1.60 ± 0.94	1.79 ± 0.78	0.10 ± 0.24	1.69 ± 1.79 *
Knee flexion	30.08 ± 7.10	36.35 ± 7.11 *	33.74 ± 6.79	39.87 ± 9.27 *
Ankle plantar flexion	−7.05 ± 7.91	−13.05 ± 7.56 *	−10.76 ± 7.52	−13.46 ± 8.57 *
Subtalar eversion	−17.42 ± 3.77	−11.50 ± 1.93 *	−2.06 ± 3.51	−13.63 ± 3.04 *

Positive values in joint angles represent the direction defined by the original angle, while negative values indicate the opposite movement. For example, a negative ankle dorsiflexion angle actually represents ankle plantar flexion. * indicates a *p*-value < 0.01 for post-intervention vs. pre-intervention data.

**Table 4:** Average muscle force of the supporting-side lower limb during single-leg jumps before and after intervention.

Muscle Force (Normalized)	Pre-CG (Mean ± Std)	Post-CG (Mean ± Std)	Pre-EG (Mean ± Std)	Post-EG (Mean ± Std)
Rectus femoris	0.83 ± 0.32	1.53 ± 0.80 *	1.77 ± 0.31	1.41 ± 0.35 *
Vastus medialis	1.00 ± 0.16	1.72 ± 0.44 *	1.00 ± 0.13	1.41 ± 0.38 *
Vastus lateralis	3.82 ± 0.71	5.23 ± 1.17 *	3.22 ± 0.38	5.33 ± 1.51 *
Biceps femoris	4.79 ± 0.82	4.63 ± 0.34	4.19 ± 0.65	2.84 ± 0.55 *
Semitendinosus	5.05 ± 0.82	5.92 ± 0.71 *	8.73 ± 4.50	2.93 ± 0.48 *
Semimembranosus	0.96 ± 0.23	1.76 ± 0.38	1.43 ± 0.59	0.51 ± 0.07 *
Gastrocnemius	9.61 ± 2.20	8.15 ± 2.38 *	7.82 ± 1.52	9.41 ± 2.35 *
Soleus	4.17 ± 0.77	4.09 ± 1.04 ^#^	3.51 ± 0.42	4.57 ± 1.18 *
Tibialis anterior	0.09 ± 0.04	0.04 ± 0.05	0.60 ± 0.42	0.25 ± 0.15 *
Gluteus maximus	1.02 ± 0.13	1.23 ± 0.26	1.10 ± 0.10	1.23 ± 0.34
Gluteus medius	1.55 ± 0.14	1.50 ± 0.30	2.09 ± 0.27	1.44 ± 0.35 *
Gluteus minimus	1.42 ± 0.19	1.11 ± 0.11 *	1.47 ± 0.34	1.03 ± 0.24 *

* indicates a *p*-value < 0.01 for post-intervention vs. pre-intervention data, and ^#^ indicates a *p*-value < 0.05, and so on.

**Table 5:** Average joint torque for the complete cycle during single-leg jumps before and after intervention.

Joint Torque (Normalized)	Pre-CG (Mean ± Std)	Post-CG (Mean ± Std)	Pre-EG (Mean ± Std)	Post-EG (Mean ± Std)
Hip abduction	0.50 ± 0.09	0.36 ± 0.14 *	0.38 ± 0.09	0.29 ± 0.10 *
Hip flexion	−1.08 ± 0.19	−1.09 ± 0.30	−0.67 ± 0.13	−1.05 ± 0.35 *
Hip external rotation	0.34 ± 0.04	0.33 ± 0.11	0.25 ± 0.05	0.27 ± 0.09 *
Knee flexion	0.10 ± 0.16	−0.16 ± 0.13 *	0.05 ± 0.07	−0.24 ± 0.17 *
Ankle plantar flexion	0.92 ± 0.22	0.92 ± 0.26	0.74 ± 0.07	0.96 ± 0.24 *
Subtalar eversion	0.23 ± 0.04	0.17 ± 0.04 *	0.10 ± 0.02	0.12 ± 0.08 ^#^

Note 2: Positive values in joint torques represent the direction defined by the original torque, while negative values indicate the opposite torque. For example, a negative hip flexion torque actually represents hip extension torque. * indicates a *p*-value < 0.01 for post-intervention vs. pre-intervention data, and ^#^ indicates a *p*-value < 0.05, and so on.

## Data Availability

Some data are contained within the article. For further detailed data, please contact the corresponding author.
